# Primary Chondroblastic Osteosarcoma of the Kidney: A Case Report and Review of Literature

**DOI:** 10.34172/aim.34620

**Published:** 2026-02-01

**Authors:** Recep Bedir, Selim Yazar

**Affiliations:** ^1^Department of Pathology, Recep Tayyip Erdogan University, Medical Faculty, Rize, Turkey; ^2^Department of Urology, Recep Tayyip Erdogan University, Medical Faculty, Rize, Turkey

**Keywords:** Chondroblastic osteosarcoma, Nephrectomy, Mesenchymal neoplasm, Renal malignancy

## Abstract

The primary renal chondroblastic variant of osteosarcoma is an exceedingly rare and aggressive malignancy with poor prognosis. This report details the case of a 40-year-old female presenting with flank pain diagnosed with primary renal osteosarcoma. We outline the clinical, radiological, and histopathological features of this tumor, along with current therapeutic strategies.

## Introduction

 Primary renal osteosarcoma is a highly uncommon mesenchymal malignancy with fewer than 40 documented cases worldwide.^1^ Due to its nonspecific symptomatology and retroperitoneal location, diagnosis is often delayed until advanced stages. Histologically, primary renal osteosarcoma is characterized by malignant osteoid-producing cells, resembling its skeletal counterpart. Extraosseous osteosarcomas account for only 1-2% of soft tissue sarcomas, with chondroblastic, telangiectatic, osteoblastic, and fibroblastic subtypes being the most prevalent. From a histological perspective, pleomorphic osteosarcoma is the most frequently reported subtype; osteoblastic and chondroblastic subtypes are rarely reported.^2^ Common clinical manifestations include a palpable abdominal mass, flank pain, and gross hematuria.^3^ Only two cases of chondroblastic primary renal osteosarcoma have been reported previously; here, we report the third case. Treatment methods such as surgery, chemotherapy, and radiotherapy have a palliative effect on this tumor. The diagnosis of primary renal osteosarcoma is based on radiological and histological findings.^1^ This report reviews the diagnostic challenges, immunohistochemical findings, and treatment approaches.

## Case Report

**Figure 1:**
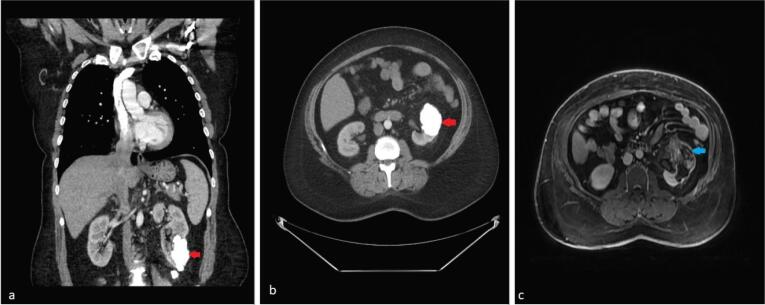
(a & b) In abdominal computed tomography (CT) with contrast enhancement, there was a calcific mass with lobulated contours, approximately 15 cm in diameter, with the widest dimensions of 7.5x5.5cm in the axial plane, associated with the left kidney, extending from the left kidney to the retroperitoneum inferiorly (red arrows). (c) In contrast-enhanced MR images, we detected a mass formation with lobulated contours, measuring 77x68x97 mm in size, originating from the lower pole of the left kidney, with T1A hypointense, T2A heterogeneous intensity, containing areas that may be due to intense calcification, and heterogeneous enhancement after IVCM, containing cystic necrotic areas, and showing restricted diffusion (blue arrow)

**Figure 2:**
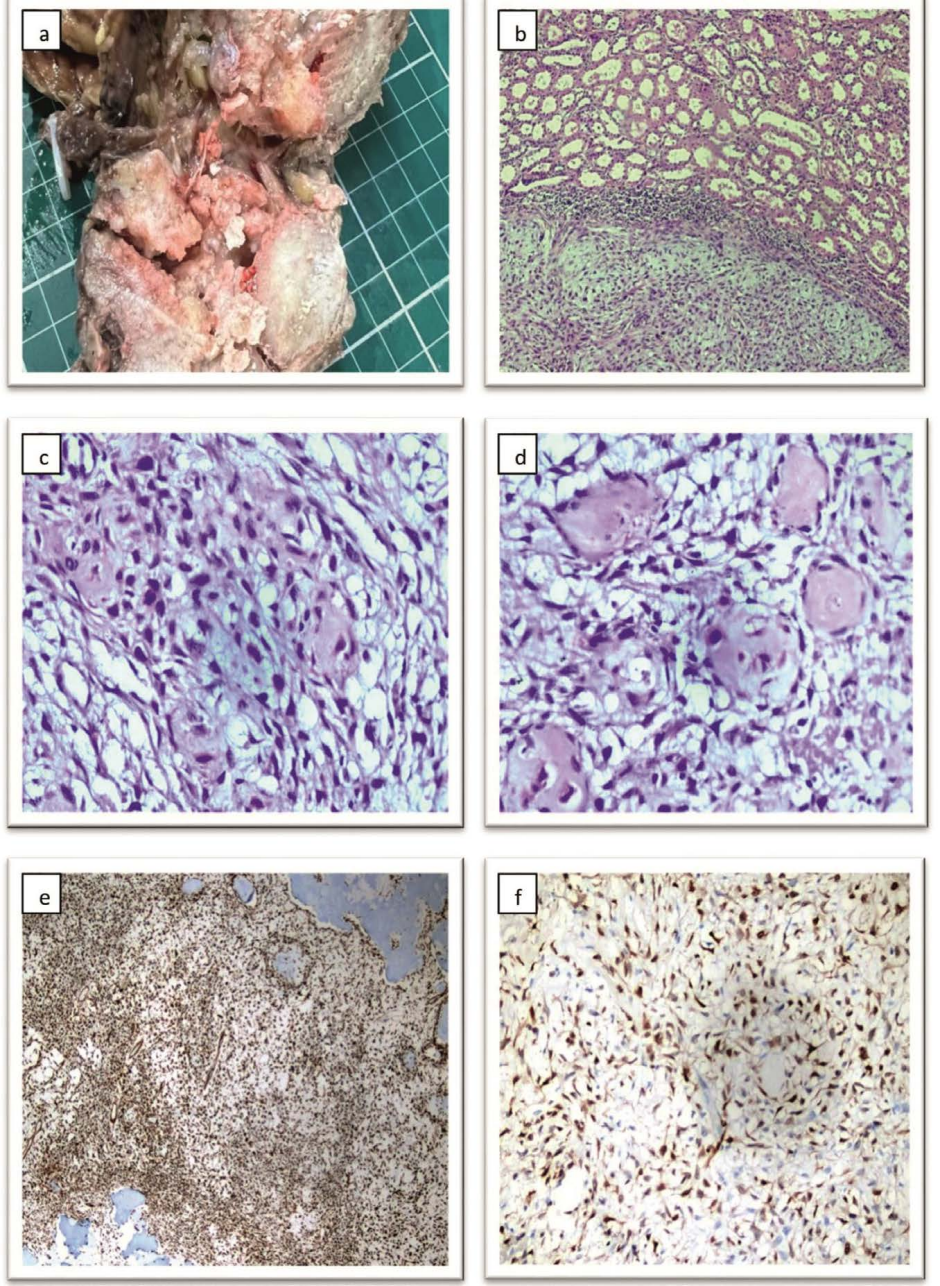
(a) Macroscopic appearance of the calcific mass. (b) Spindle cell tumor adjacent to the renal parenchyma. (H&E x40). (c) The tumor consists of atypical spindle cells with large hyperchromatic nuclei and prominent nucleoli. (H&E x400). (d) Osteoid formation surrounded by atypical spindle cells was observed in the tumor. (H&E x400). (e) Diffuse positive staining with vimentin was observed in the tumor (x100). (f) Weak positive staining with S100 was observed in the tumor (x200)

 A 40-year-old woman presented to the internal medicine out-patient clinic with flank pain. On physical examination, a hard mass was palpated in the left mid-abdominal quadrant. Routine urine and blood tests were normal. However, a left renal mass was reported on abdominal ultrasound, prompting consultation with the urology department. Contrast-enhanced abdominal computed tomography (CT) revealed a calcified left renal mass (Figure 1a, 1b). Subsequent magnetic resonance imaging (MRI) demonstrated a heterogeneous solid-cystic lesion (Figure 1c). After the uroradiooncology council, a tru-cut biopsy was considered because the mass was atypical and extemely rare, but it was eventually decided that a biopsy could not be taken due to severe calcification and solidity, and a radical nephrectomy decision was made with an initial diagnosis of a left malignant renal mass. The giant mass associated with the left kidney was excised using a laparoscopy-assisted radical nephrectomy technique, including the pararenal fat tissues. On gross pathological examination, the left radical nephrectomy specimen weighed 900g and measured 17x9x9 cm. A bone-hard mass measuring 12x8x6 cm was identified in the lower pole. The tumor grossly filled the pelvicalyceal system and infiltrated the perirenal fat tissue and renal sinus fat tissue (Figure 2a). Microscopically, the tumor caused atrophy of adjacent renal tissue, contained widespread dystrophic calcifications, and formed bone trabeculae (Figure 2b). The tumor was composed of atypical spindle cells with chondrosarcoma-like differentiation, occasionally forming cellular areas within a myxoid matrix (Figure 2c). In our case, widespread bone formation and homogeneous oscification with chondroblastic or osteoblastic morphology and widespread irregular calcifications were seen. Mild to moderate pleomorphisms and low mitotic activity were observed. Globular and ovoid neoplastic osteoid formations were also noted (Figure 2d). No tumor was observed at the surgical margins and ureteral surgical margin. Renal vein invasion and necrosis were not observed in the tumor. In immunohistochemical examination, positive staining with vimentin, S100, and negative staining with pan-CK (cytokeratin), PAX8, desmin and CD99 were found in the tumor (Figure 2e, 2f). In the differential diagnosis of dedifferentiated liposarcoma with extensive osseous metaplasia, no fatty component was observed on microscopic examination of the tumor. In the immunohistochemical examination performed for the differential diagnosis of dedifferentiated liposarcoma with extensive osseous metaplasia, positive staining with CDK4 and MDM2 was not observed. Additionally, CDK4/MDM2 amplification was not observed in the FISH study. The possibility of sarcomatoid renal cell carcinoma was excluded due to pan-CK and PAX8 negativity in the tumor and the absence of an epithelial component in microscopic examination. To exclude leiomyosarcoma, which is a common sarcoma in the kidney, desmin was found to be negative in immunohistochemical examination. The Ki-67 proliferation index was low (3-4%) in the tumor. After excluding other tumors in the differential diagnosis with immunohistochemical and molecular studies, a diagnosis of chondroblastic osteosarcoma was made. The pathological stage of the tumor was reported as PT3aN0M0. The patient received four cycles of postoperative chemotherapy (doxorubicin + ifosfamide), and no recurrence or metastasis was detected after one year of follow-up. Written informed consent was obtained from the patient.

## Discussion

 Sarcomas represent only 1% of renal malignancies and tend to have a worse prognosis compared to other renal cancers.^2^ Sarcomas are rare tumors in adults including renal synovial sarcomas and account for only 1% of primary renal malignancies. Leiomyosarcomas have the highest prevelance. Renal metastasis of osteogenic sarcoma occurs in up to 13% of primary skeletal osteosarcomas.^4^ The “sunburst” appearance is relatively characteristic on a CT scan. Primary renal osteosarcoma is a rare sarcoma characterized by calcium deposition and associated with worse outcomes compared to other renal tumors. Flank pain, gross hematuria, and weight loss are the most common presenting symptoms. Microscopic examination of the tumor shows osteoid formation within a spindle cell proliferation. The sarcomatoid variant of renal cell carcinoma (RCC) is an important differential diagnosis of renal osteosarcoma. The presence of epithelial cell tumor in histology is indicative of the sarcomatoid variant of RCC.^5^ Pleomorphic osteosarcoma is the most frequently documented subtype, whereas osteoblastic and the chondroblastic forms are less common.^6^ The tumor is difficult to detect due to its location in the abdomen and lack of specific findings. Metastasis and local recurrence are common in extraskeletal osteosarcoma, with some patients presenting with metastatic disease. The literature reports that tumor metastasis most commonly spreads to the lungs. Other metastatic sites include the soft tissue, bone, peritoneum and lymph nodes.^7^ These tumors are often diagnosed at an advanced stage, with 32% of patients presenting with metastases.^8^ Given their aggressive nature, early detection is crucial. Surgery alone may be sufficient for low-grade tumors limited to the kidney. For distinguishing from dedifferentiated liposarcoma with extensive osseous metaplasia, CDK4/MDM2 positivity was not observed in both immunohistochemical and molecular studies. Sarcomatoid renal cell carcinoma with widespread foci of osseous metaplasia was ruled out due to pan-CK and PAX8 negativity.

 The etiology of renal osteosarcomas remains unclear. Based on Virchow’s theory, exposure to X-rays and radioactive materials are considered potential risk factors. Under certain circumstances, such as radiation exposure, the connective tissue may undergo metaplastic transformation into embryonic mesenchyme, which can subsequently differentiate into osteoblasts and form bone.^4^

 To date, 31 primary renal osteosarcomas have been documented in the literature.^1^ The presentation in our patient, particularly the imaging and pathological findings, shares similarities with previous reports yet also shows distinct differences, highlighting the heterogeneity of this tumor. Unlike previously reported cases, our case was a low-grade chondroblastic osteosarcoma, the least frequently reported in the literature. Flank pain, which was also observed in the present study, is the most common presenting symptom of renal osteosarcoma. Radiological imaging of the tumor showed a sunburst appearance due to intense calcification and ossification.

 Management of renal osteosarcoma requires a multimodal strategy incorporating surgical resection, systemic chemotherapy, and radiotherapy. Surgical intervention remains the cornerstone of treatment, with wide local excision and negative margins being essential for optimal oncologic control. Current evidence, though limited by the rarity of this malignancy, supports the use of platinum-based adjuvant chemotherapy regimens similar to those employed in extraosseous osteosarcoma. The combination of doxorubicin, ifosfamide, and cisplatin has emerged as a particularly effective protocol, demonstrating superior outcomes compared to historical monotherapy approaches.^9^ Recent advances have further shown that incorporating targeted agents such as Anlotinib with conventional chemotherapy may significantly prolong disease-free survival.^10^

 Radiotherapy plays a distinct role in the therapeutic arsenal, particularly for local disease control. Available data suggest that radiation therapy may be more effective than chemotherapy alone in preventing local recurrence, and its concurrent administration with systemic therapy appears to yield survival benefits over surgical intervention alone.^11-12^ In the present case, the patient received four cycles of adjuvant combination chemotherapy (doxorubicin, ifosfamide combination) following nephrectomy. Standard care of treatment in soft tissue sarcoma is unclear. The benefit of adjuvant treatment is not demonstrated with highly scientific evidence. In this case, we used dual chemotherapy despite the low proliferation index. We did not prefer cisplatin in this case because of the renal toxicity of platin regimens and the low Ki-67 proliferation index. According to oncology guidelines, one can choose ifosfamid or cisplatin-based treatments in extraskeletal osteosarcoma.

 Notably, emerging immunotherapeutic approaches such as PD-1 inhibition have shown preliminary promise in managing osteosarcoma progression, though comprehensive clinical validation remains pending.^10^ This underscores the need for further investigation into novel treatment modalities for this aggressive malignancy.

 We summarize the clinical features, pathological findings, demographic characteristics, therapeutic approaches, and clinical outcomes of previously reported renal osteosarcoma cases along with our current case in Table 1.

**Table 1:** Summary of Clinical and Pathologic Features of 32 Primary Renal Osteosarcoma Cases.

**Reference**	**Age/gender**	**Presenting symptoms**	**Specimen laterality**	**Treatment**	**Outcome**	**Tumor size**	**Metastases**	**AJC stage**
Haining.^8^ (1936)	76/Male	Hematuria	Left	None	Not mentioned	Not mentioned	Liver, bowel, and right kidney	IV
Hamer and Wishard.^13^ (1948)	76/Male	Hematuria	Right	Radiotherapy	1 month	Not mentioned	Lung	IV
Hudson.^14^ (1956)	52/Female	Flank pain and hematuria	Left	Radical nephrectomy	Died 4 months after surgery	Not mentioned	Transvers colon	?
Soto *et al.*^15^ (1965)	82/Female	Flank pain, gross hematuria	Left	Radical nephrectomy	Died 82 days after surgery	15x14x9 cm	Lungs, liver and omentum	IV
Johnson *et al*.^16^ (1970)	59/Female	Flank pain, nausea and vomiting	Right	Radical nephrectomy	Died 17 days after surgery	5x4 cm	Local metastasis	II
Chambers and Carson^17^ (1975)	43/Male	Flank pain	Left	Radical nephrectomy	Died 9 months after surgery	Not mentioned	Liver	IV
Axelrod *et al*.^18^ (1978)	48/Male	Abdominal distension, weight loss and diarrhea	Right	None	Died 1 year after tumor discovered	2200 (no size given)	Liver, spleen, bone marrow and lungs	IV
Biggers and Stevart.^19^ (1979)	67/Male	Physical examination	Right	Open biopsy	Died 4 months after surgery	23 cm	Lungs	IV
Bollack *et al*.^20^ (1982)	29/Male	Abdominal pain, anorexia and weight loss	Left	Radical nephrectomy, chemotherapy	No abnormality 6 months after surgery, with local recurrence and distant metastases 3 weeks later, died 10 days later	17x12x10 cm	Pancreas	IIIB
Micolonghi et al.^21^ (1984)	48/Female	Flank pain	Right	Laparotomy, chemotherapy	Died 4 weeks after surgery	12.5 cm	Lungs	IV
Mortensen *et al*.^22^ (1989)	56/Male	Flank pain, gross hematuria	Right	Radical nephrectomy, chemotherapy	Died 18 months after surgery	4 cm	Lungs metastasis, 7 months after surgery	IA
Acha Perez *et al*.^23^ (1993)	47/Male	Gross hematuria and left back pain	Right	Laparotomy failed to excise	Died 3 months after surgery	8x8 cm	Local metastasis	IIIB
Ah-chong and Yip^24^ (1993)	56/Male	Abdominal pain	Right	Radical nephrectomy	Metastasis 6 months after surgery	Not mentioned	Bone metastasis 6 months after surgery	?
Weingartner *et al*.^25^ (1995)	48/Male	Flank pain, gross hematuria	Left	Radical nephrectomy, chemotherapy	Died 15 months after surgery	Not mentioned	Local recurrence and distant metastasis to pleura and liver	
Watson *et al*.^5^ (1995)	47/Male	Abdominal pain, loss of appetite and lethargy	Right	Radical nephrectomy	Died 4 months after surgery	12x9x9 cm	Invasion of liver capsule	IIIB
Messen *et al*.^26^ (1995)	46/Male	Not mentioned	Left	Radical nephrectomy	Disease-free 16 months	15x9x8 cm	Left adrenal gland	IIIB
Leventis *et al*.^27^ (1997)	67/Male	Flank pain, gross hematuria	Left	Radical nephrectomy, regional lymphadenectomy, chemotherapy	Died 4 months after surgery	28x14x10 cm	Lungs metastasis 6 weeks after surgery	IB
Ito *et al*.^28^ (1997)	67/Female	Physical examination	Left	Radical nephrectomy	Recurrent 2 months after surgery. Died 4 months after surgery	25x18x15 cm	Local metastasis, 2 weeks after surgery	IB
Leggio et at.^29^ (2006)	60/Male	Abdominal pain	Left	Radical nephrectomy	Died 8 months after surgery	12 cm	Local recurrence, 7 months after surgery	IB
Tommaso *et al*.^30^ (2007)	79/Male	Flank pain, weakness and loss weight	Left	Radical nephrectomy, radiation therapy	Died 7 months after surgery	22x16 cm	Local recurrence and distant metastasis to diaphragm, pleura and ribs, 3 months after surgery	IIIB
Puri *et al*.^31^ (2012)	65/Female	Flank pain, gross hematuria and occasional dysuria	Left	Radical nephrectomy, chemotherapy	Not mentioned	Not mentioned	Lungs and bladder metastasis, 1 months after surgery	?
Lopez-Beltran *et al*.^6^ (2014)	50/Female	Pelvic and back pain	Left	Radical nephrectomy	At least 6 years	5.5x4.9 cm	None	IB
	66/Female	Back pain	Left	Radical nephrectomy	At least 2 years	3.5x3.2x3.2 cm	None	IA
	78/Female	Flank pain, gross hematuria	Left	Radical nephrectomy	Died 14 months after surgery	7x6x5.1 cm	Lungs and brain metastasis, 1 year after surgery	IB
Flynn *et al*.^32^ (2015)	77/Female	Gross hematuria and flank pain	Left	Radical nephrectomy	Disease-free at least 2.5 years after surgery	3.3 cm	None	IA
Virgilio *et al*.^3^ (2017)	59/Female	Abdominal distension and intestinal subocclusion	Left	Radical nephrectomy	Died 3 months after surgery	15 cm	Left colon and adrenal gland	IIIB
Zhang *et al*.^9^ (2024)	41/Male	Flank pain	Right	Radical nephrectomy, chemotherapy	Disease-free at least 8 months	10x9x8 cm	None	IB
Huang *et al*.^8^	48/Female	Abdominal distension and gross hematuria	Left	Radical nephrectomy, chemotherapy	Disease-free at least 26 months	21x18x11 cm	Lungs	IV
Namdari *et al.*^1^ (2022)	43/Female	Flank pain, gross hematuria, weakness and weight loss	Left	Radical nephrectomy, chemotherapy, radiotherapy	Disease-free at least 20 months	15x10x8.5 cm	None	IIIA
Chen *et al*.^4^ (2023)	54/Female	Abdominal pain and loss weight	Left	Lesion excision	Disease-free at least 13 months	8x6x5 cm	None	?
Su *et al*.^10^	63/Male	Left side flank pain and gross hematuria	Left	Radical nephrectomy	Died 18 months after surgery	9x7x7 cm	Descending colon mesentery and lung	?
Present Case	40/Female	Flank pain	Left	Radical nephrectomy +chemotherapy	Disease-free at least 1 year	12x8x6 cm	None	IIIA

## Conclusion

 Renal osteosarcomas are rare and aggressive tumors with poor prognosis. They should be included in the differential diagnosis of all renal masses. Early diagnosis is so essential for the patient’s treatment, prognosis and recovery.
